# Food Prices, Ethics and Forms of Speculation

**DOI:** 10.1007/s10551-021-04842-z

**Published:** 2021-05-23

**Authors:** Don Bredin, Valerio Potì, Enrique Salvador

**Affiliations:** 1 College of Business, University College Dublin, Blackrock, Dublin Ireland; 2 Universitat Jaume I, Castelló de la Plana, Spain

**Keywords:** Commodity prices, Financialization, Commodity speculation, Q49, G12, G15

## Abstract

This paper examines the role of speculative motives in the determination of commodity prices and specifically food related commodity prices. The motivation for this study is the considerable flow of funds into commodities, the widespread view that the process of financialization has led to greater levels of speculation and that speculation is the primary cause of regular spikes in food prices since the turn of the century. We consider two forms of short-term trading, a biasing influence (Manipulators) and a correcting influence (Speculators), relative to the fundamental price. While both forms of short-term trading are relevant, they are small in terms of their influence on overall prices. We do however find some evidence of an increased role being played by Manipulators during the period most associated with financialization.

## Introduction

A regular commentary, during the commodities super cycle of the early to mid 2000s, was the *obvious* role played by financialization and, by extension, speculators. References to commodities such as oil and in particular the food versus fuel debate were to the fore during this period. High-profile examples include the Time magazine article entitled “Betting on Hunger: Is Financial Speculation to Blame for High Food Prices?” (Paramaguru, 2012), highlighting the influence of financial speculators on food prices. While the article did provide a brief discussion of possible fundamental causes such as the weather, non-OECD demand and biofuel policy, the main focus was on the role of commodity speculation on food prices. Testimony to the US Congress by hedge fund manager (Masters, 2008) also highlighted this issue. In particular, Masters (2008) maintained that growth in index investment created a bubble in commodity future prices which then fed through (via arbitrage) to spot prices. However, compelling the argument by Masters (2008), most academic studies have been unable to find a direct link between index trading and commodity futures price movements or the physical prices for that matter. Irwin and Sanders (2011), Cheng and Xiong (2014) and Haase et al. (2016) all provide excellent reviews of the literature.^1^

To explain commodity prices within the food sector, we examine the role of a combined set of actors or investors. We distinguish between long-term (LT) investors, namely those focusing on fundamentals, and short-term (ST) investors. We consider two types of ST investors, Speculators and Manipulators. Our model develops an empirical application of Angel and McCabe (2009) description of speculation and manipulation in commodity markets. We consider the case of ST investors operating on legitimate grounds, who attempt to benefit from price changes (*Speculators*) and those that actively engage in pushing prices away from their fundamental value (*Manipulators*).^2^ Both take a short-term horizon, however, the Speculators act rationally (explained in detail in the next paragraph), while the Manipulators take a contrarian focus. We also examine the case of agents that are only interested in long-run fundamentals (*Fundamentalists*). Rather than examining each case separately, we simultaneously combine all three agents in relation to commodity price movements. Evidence consistent with the popular narrative described above would lead to a definitive increase in the role of Speculators or Manipulators since the period of financialization.

At the heart of our classical fundamental-based model, lies the rational asset pricing model (RAPM). The RAPM indicates that commodities are valued based on their *cash flows* (convenience yield) that are likely to be generated, see Bredin et al. (2018). We refer to this price as the price determined by the long-run actors, the Fundamentalists. As highlighted above, we will also examine Speculators and Manipulators, using our ethical motivation. As a sensitivity test we compare our results with a purely data driven proxy for the role of Fundamentalists, Speculators and Manipulators using data from the ‘Commitment of Traders’, which is sourced from the Commodities Futures Trading Commission.

Our results provide consistent evidence that the price of commodities within the food sector are certainly influenced by the behaviour of both Speculators and Manipulators. In particular we find that commodity prices are far better explained by simultaneously taking account of fundamentals and both speculation and manipulation. The popular narrative described above might interpret this result as being a recent phenomenon and driven by the process of financialization. However, our findings highlight that this role exists before any financialization process began. While the importance of Speculators/Manipulators are certainly relevant, the vast majority of the post 1990 sample is driven by Fundamentalists. We do, however, find evidence that while the role of Manipulators is small, their importance has grown during the period most associated with financialization.

## Commodity Prices, Ethics, Financialization and Speculation

A commodity can be defined as a raw material or a primary agricultural resource, that can be purchased or sold for production and/or consumption. Most references to commodities over the last decade, however, have in reality viewed them in a different light. In particular, rather than as a *consumption asset*, commodities have hit the headlines as an *investment asset*. The flow of investors’ funds into commodities since 2000 has been striking, with the $200 billion in 2008 growing to $380 billion by 2011, see Rouwenhorst and Tang (2012). In 2018, commodity derivatives globally represent close to $2 trillion of outstanding over the counter (OTC) derivatives.^3^ Investors’ activity sparked considerable interest from the academic literature, with a flood of empirical evaluations on the benefits of commodity investing. The benefits of commodity investing, as discussed by Gorton and Rouwenhorst (2006) and more recently by Bhardwaj et al. (2015), are believed to include consistent risk adjusted returns and low correlations relative to US equities.^4^

The flood of funds into commodities, via commodity futures, has been referred to as the financialization of commodities. This process represents a structural change in commodity market participation and, combined with the price increase during the super cycle, leads to concerns over the potential role of speculation. Parts of the media and, to some extent, regulators have adopted the view that this process of financialization has been feeding through to physical commodity prices, to such an extent that physical prices are no longer closely tied to their fundamental value. Formal evidence of the increased role of financialization since 2005 has been provided by Tang and Xiong (2012) and most recently Bhardwaj et al. (2015).^5^ Further evidence is provided by Büyükşahin and Robe (2011, 2013) and Silvennoinen and Thorp (2013), who show that the return correlation between commodities and conventional financial assets (stocks) has turned positive and statistically significant since 2008, after being negative and significant in previous years. While Cheng and Xiong (2014) examine the implications of financialization in relation to both risk sharing and price discovery, Gilbert (2010) highlights the process of financialization as the principle cause of food commodity price rises during the super cycle.

The natural interpretation is and has been that the process of financialization will lead to greater levels of speculation within commodities. This interpretation very much relies on speculation forcing prices away from their fundamental values and so might be refereed to as manipulation following Angel and McCabe (2009). To date, there has been no formal empirical analysis of the role of both forms of short-run behaviour in relation to commodity price movements. There is, however, considerable evidence in favor of fundamentals driving recent movements in commodity prices and oil prices in particular. For the case of a broad range of commodities, Sanders and Irwin (2011) find evidence against speculative effects and consistent with fundamentals. Andreasson et al. (2016) also provides evidence against the link between speculation and food prices. Smith (2009) finds no empirical evidence to indicate that speculation increased oil prices, while Juvenal and Petrella (2015) find that speculation as well as economic fundamentals played a significant role in the oil price increase during the 2004–08 period. Hamilton (2009a, 2009b) provides a comprehensive overview of the potential causes of oil price changes and he concludes that speculation contributed to the price rise. Finally, Knittel and Pindyck (2016) find that speculation certainly played a role in the 2004-08 oil price increase, but was not the only contributor of the price increases. Our study is novel in the literature, as we examine for the first time the simultaneous role of fundamentals, speculation and manipulation in relation to food based commodities, and with a particular focus on financialization.

**Table 1:** Long-term vs. short-term traders

Statistic (*p*-value)	Corn	Oats	Soybeans	Soybean oil	Wheat	Coffee
$H_0: \mu _{LT}= \mu _{ST}$	27.0644***	28.2277***	20.1624***	92.8960***	29.9230***	48.1897***
	(0.0000)	(0.0000)	(0.0000)	(0.0000)	(0.0000)	(0.0000)
$H_0: \mu _{LT} < \mu _{ST}$	27.0644***	28.2277***	20.1624***	92.8960***	29.9230***	48.1897***
	(0.0000)	(0.0000)	(0.0000)	(0.0000)	(0.0000)	(0.0000)

This table shows the statistic and the *p*-values of a two-tailed (top row) and one-tailed (bottom row) equality mean tests between the fundamental weights from the behavioral model (long-term traders) and the sum of Speculators and Manipulators weights from the behavioral (short-term trader) for each commodity

***, ** and * represents rejection of the null hypothesis at 1%, 5% and 10% significance level

**Table 2:** Manipulators and pairwise commodities

Statistic (*p*-value)	Corn	Oats	Soybeans	Soybean oil	Wheat
Oats	− 0.5673				
	(0.5707)				
Soybeans	− 2.1362	− 1.4714			
	(0.0331)	(0.1417)			
Soybean oil	9.2202***	8.4544***	8.8897***		
	(0.0000)	(0.0000)	(0.0000)		
Wheat	1.0029	1.3435***	2.7367*	− 8.0542***	
	(0.3163)	(0.0000)	(0.0064)	(0.0000)	
Coffee	11.9795***	10.9138***	11.0849***	2.5313	10.7384***
	(0.0000)	(0.0000)	(0.0000)	(0.0116)	(0.0000)

This table shows the statistic and the *p*-values of a two-tailed equality mean tests between the weights of the Manipulator agent from the behavioral model for each pair of commodities. For instance, the intersection between the corn column and the oats row shows the test results for the Manipulator agent weights of corn and oats

***, ** and * represents rejection of the null hypothesis at 1%, 5% and 10% significance level after Bonferroni correcting the significance levels for multiple comparisons

## Commodity Prices: A Behavioural Setting

Our starting point is the present value model of asset prices, with a specific focus on commodity prices. Commodity prices can be defined as the present value of expected future ’payoffs’ associated with holding the commodity.

1 $$\begin{aligned} P_t = \sum ^\infty _{i=1} \delta ^i E_t \psi _{t+i} \end{aligned}$$

Following Pindyck (1993), commodity prices ($P_t$) will change when there is changes in expected future ’payoffs’ and/or changes to the discount factor ($\delta$). The term $\delta = \frac{1}{1+\mu }$, where $\mu$ represents the expected return to holding the specific commodity. The normal application in equity markets would adopt the cash flows (dividends) as the payoffs. However, drawing on the theory of storage, these payoffs are defined as the benefits that accrue to the holder of a storable physical commodity, rather than the owner of the futures contract (see Bredin et al., 2018). These benefits are referred to as the convenience yield ($\psi$) and represent the benefits associated with holding inventory, e.g. maintaining a smooth production process and so avoiding any disruption to the flow of goods being produced. The market price of all commodities (e.g. agricultural and energy) will be determined by the expectation of market scarcity, reflected in the interaction between current supply and demand. The above present value model can be interpreted as a reduced form dynamic demand-supply model of commodity prices. The implication is that even when the expected capital return is small (relative to the risk adjusted rate) or negative, firms will still maintain positive stocks of inventory. Although formally the convenience yield is unobserved, an indication can be determined via an arbitrage relationship between spot prices and futures prices.^6^ See Bredin et al. (2018) for a detailed discussion.

A simplified version of the model is described below in graphical terms in the upper part of Exhibit 1. Rather than focusing on prices, for modelling reasons we examine the differential form of Eq. 1. The percentage net basis (PNB) is defined as the ratio of the convenience yield to price. This is consistent to the dividend price ratio when examining equities. On the vertical axis the relative value of the commodity, the (normalized) PNB, is reported. The time horizon in years is indicated on the horizontal axis, going from 1 year to 100 years (proxying the infinite investment horizon in the future). The relative value of the commodity today is determined by some combination of expected next period prices (*diagonal pattern*) and the present value of the convenience yield (*grey*).

**Exhibit 1:**
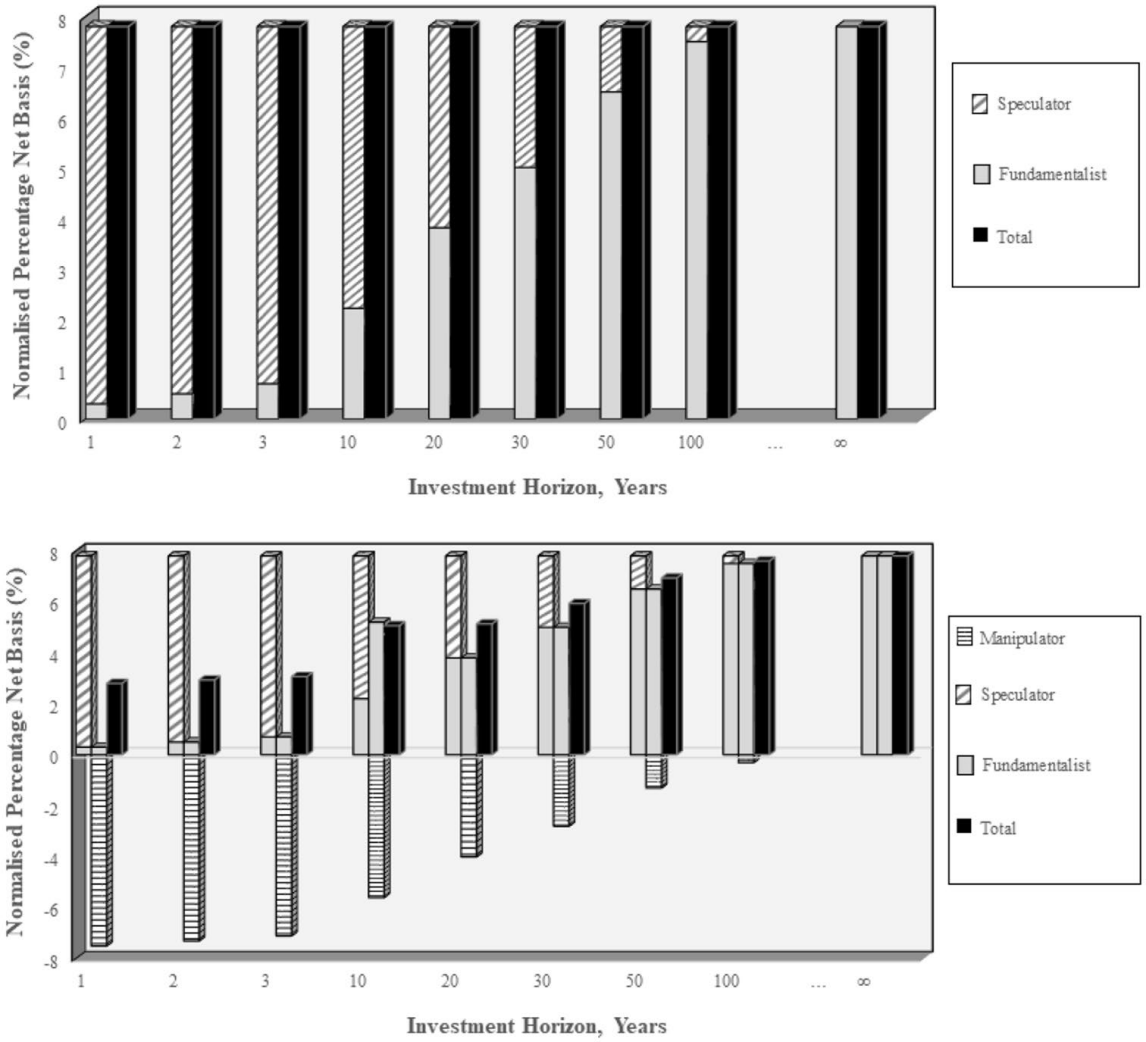
Commodity pricing expectations

The RAPM reported in Eq. 1, is represented by the commodity price being determined by an infinite stream of future payoffs associated with the physical commodity, reflected on the far right hand side of the figure. As the horizon grows, the importance of the convenience yield (*grey*) in relation to the relative commodity value increases in importance. Equation 1 reflects the case with infinite horizon and is consistent with the grey bars in the exhibit. This reflects the LT investor or Fundamentalist. Rather than focusing purely on the long-term, we could also examine a short-term perspective, the ST investor. We first consider the ST investor with a rational perspective. In the one period case, today’s relative value are primarily determined by expected one period returns (*diagonal pattern*). This is a perfectly rational setting, with the only caveat being that the investment horizon is short-term. We refer to this ST investor setting as the *Speculator*. As the investment horizons increase in time, the relative value of the commodity is increasingly driven by the fundamental element (or the convenience yield) and less by the one period return component. Critically both indicators of relative commodity value are consistent, as we move through time. The key take away from the exhibit is that commodity values will be consistent, irrespective of whether prices are driven by fundamentals (*grey*) or speculation (*diagonal pattern*).

Finally, we also consider the case of the agent that actively engage in pushing prices away from their fundamental value (*Manipulators*). Angel and McCabe (2009) define manipulation ‘as taking actions designed to move prices away from prices justified by economic fundamentals and to profit from the disruption’ (p. 282). This definition places considerable emphasis on the unethical aspect of this behaviour. A consistent interpretation, which also encompasses our model, is that this behaviour is primarily irrational. This may be as a result of herd behaviour following an announcement, e.g. an over-reaction to poorer than expected crop yields. The critical element in relation to manipulation is that prices are being pushed away from their fundamental value. Rather than the combined actions resulting in stable long-run prices, *Manipulators* push prices higher or lower with the resulting relative price not converging to a stable level. The case of Fundamentalists, Speculators and Manipulators are presented in the lower part of Exhibit 1, with the role of Manipulators reflected in the horizontal patterns. Rather than the one period and the infinite horizon being perfectly consistent, we now see that Manipulators have an adverse impact on relative value, in particular at the short horizon. Our aim is not to formally test for financialization in food commodities. We are, however, interested in examining whether the conventional view of financialization inspired manipulation is present and a driving force for food commodities. Rather than focusing on an individual actor, in our empirical analysis we simultaneously model Fundamentalists, Speculators and Manipulators, with the fraction assigned to each actor being determined by the relative predictive performance.

Following Brock and Hommes (1998) and, more recently, Lof (2015), we aggregate the three agents in a heterogeneous agent model in which the PNB, $y^{T}_t$, is determined as follows;

2 $$\begin{aligned} y_t^{T} = G^{F}_t y^{'F}_t + G^{S}_t y^{'S}_t + G^{M}_t y^{'M} \end{aligned}$$

Here, the term $G^j$ represents the fraction of the PNB determined at the margin by each agent (Fundamentalists (*F*), Speculators (*S*) and Manipulators (*M*)).^7^ The fraction assigned to each agent increases when the agent’s prediction outperform the others’, and the fractions sum to unity, as described by the following multinomial logit model;

3 $$\begin{aligned} \begin{aligned} G^j_t&= \frac{exp(\omega ^j U^j_t)}{\sum {_k} exp(\omega ^k U^k_t)} ~ ~ j,k \in {F, S, M} \\ where ~ U^j_t&= -(y'^{j}_{t-1} - y'_{t-1})^2 ~ ~ j \in {F, S, M} \end{aligned} \end{aligned}$$

The $\omega ^j$ parameters represent the willingness of agents to switch their strategy. A higher $\omega ^j$ indicates greater willingness to switch. The term $U^j_t$ represents a measure of fit, defined as the squared difference between the theoretical and the actual normalized PNB. Following the original recommendation of Hong et al. (2007) and the subsequent application by Lof (2015), we allow each $\omega ^j$ to vary depending on the agent.

In terms of formal tests we examine three particular hypothesis. Our first hypothesis test will examine whether the combined role of speculation and manipulation outweighs the role played by fundamentals over the course of our sample. Although Speculators in our model represent price correcting agents, we adopt a *conservative* setting, where we evaluate the role of both Speculators and Manipulators versus Fundamentalists.

### Hypothesis 1:

Short-term trading (speculation and manipulation) outweighs long-term trading.

Our second and third hypotheses test examine the extent of systematic manipulation across our commodities and over time. If financialization-inspired manipulation exists, we should see some systematic behaviour for manipulation in the latter half of our sample. To examine the nature of manipulation, we plot the share of manipulation across all commodities. The literature to date has highlighted early to mid 2000s as the likely start of the financialization process within commodities. For example, Hamilton and Wu (2014), Cheng and Xiong (2014) and Chari and Christiano (2017) point to 2005, 20004 and 2002, respectively.^8^ The extent of systematic manipulation at the commodity level and the implications of financialization (if any) are tested using the following two hypotheses.^9^

**Table 3:** Financialization and manipulation

Statistic (*p*-value)	Corn	Oats	Soybeans	Soybean oil	Wheat	Coffee
$H_0: \mu ^{pre02}_{M}= \mu ^{pos02}_{M}$	0.0376	$-1.0507$	5.5964***	$-0.7968$	$-2.1867$**	$-0.0085$
	(0.9701)	(0.2942)	(0.0000)	(0.4262)	(0.0295)	(0.9993)
$H_0: \mu ^{pre02}_{M}< \mu ^{pos02}_{M}$	0.0376	$-1.0507$	5.5964***	$-0.7968$	$-2.1867$	$-0.0085$
	(0.4850)	(0.8529)	(0.0000)	(0.2131)	(0.9852)	(0.5034)

This table shows the statistic and the *p*-values of a two-tailed equality mean tests between the weights of the Manipulator agent from the behavioral model before 2002 and the weights of the Manipulator agent from the behavioral model after 2002 for each commodity

***, ** and * represents rejection of the null hypothesis at 1%, 5% and 10% significance level

**Table 4:** Long-term vs. short-term traders—CFTC

Statistic (*p* value)	Corn	Oats	Soybeans	Soybean oil	Wheat	Coffee
$H_0: \mu _{LT}= \mu _{ST}$	39.2842***	55.7041***	35.8138***	34.7978***	19.8876***	32.2064***
	(0.0000)	(0.0000)	(0.0000)	(0.0000)	(0.0000)	(0.0000)
$H_0: \mu _{LT} < \mu _{ST}$	39.2842***	55.7041***	35.8138***	34.7978***	19.8876***	32.2064***
	(0.0000)	(0.0000)	(0.0000)	(0.0000)	(0.0000)	(0.0000)

This table shows the statistic and the *p*-values of a two-tailed (top row) and one-tailed (bottom row) equality mean tests between the fundamental weights obtained from CFTC data (long-term traders) and the sum of Speculators and Manipulators weights obtained from CFTC data (short-term trader) for each commodity

***, ** and * represents rejection of the null hypothesis at 1%, 5% and 10% significance level

### Hypothesis 2:

Manipulation is systematic across all commodities.

### Hypothesis 3:

Manipulation is distributed consistently across our full sample.

## Data and Empirical Results

### Data

Our study examines a total of 6 food commodities covering corn, oats, soybeans, soybean oil, wheat and coffee using monthly data over the period 1990 to 2017. Exact details on each contract and sample coverage are reported in Appendix 2.^10^ Data is sourced from the Commodity Research Bureau (CRB) and the Commodities Futures Trading Commission (CFTC). All reported data is for futures prices on the first Wednesday of each month, Tuesday prices were adopted when the Wednesday price is not available. The proxy for the spot price ($P_t$) is the spot futures contract, i.e. the contract expiring in month *t*. The proxy for the futures price ($F_t$) will then be the next to expire futures contract. Thus the spot and futures prices represent the exact same good and the time intervals between the two delivery dates is known.

**Fig. 1:**
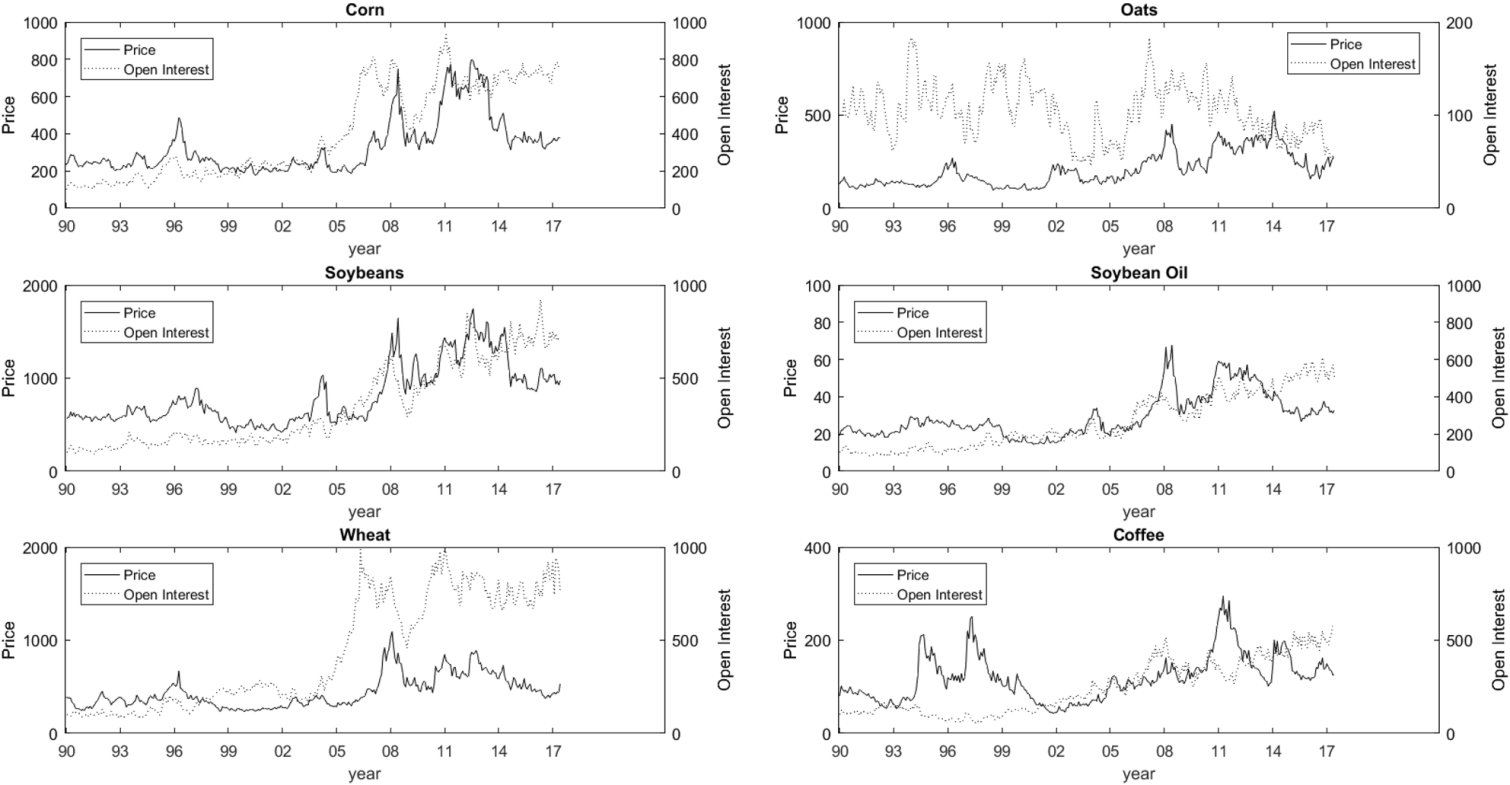
Commodity prices and open interest

The process of financialization has been particularly prevalent in relation to a particular class of investors, commodity index traders (CITs). The CIT’s represent investors, that do not hold the physical commodity, but hold commodity futures as part of a larger portfolio strategy.^11^ Cheng and Xiong (2014) provide an excellent summary of the financialization process and the importance of CIT’s. The process of financialization has meant that gross positions in commodity futures markets have increased dramatically. The most recent evidence, provided by Chari and Christiano (2017), has indicated that the financialization process began in 2002. Both commodity prices (solid line) and open interest (broken line), representing gross positions, are reported in Fig. 1. While there certainly were some spikes in prices throughout the 1990s, the large increase tends to occur around the early 2000s, in particular around 2004/2005. This is also the time when open interest began to increase dramatically. There certainly appears to be graphical justification to link financialization with the price increases on the early 2000s. Following Chari and Christiano (2017) we take 2002 as the starting point of the financialization process.

**Fig. 2:**
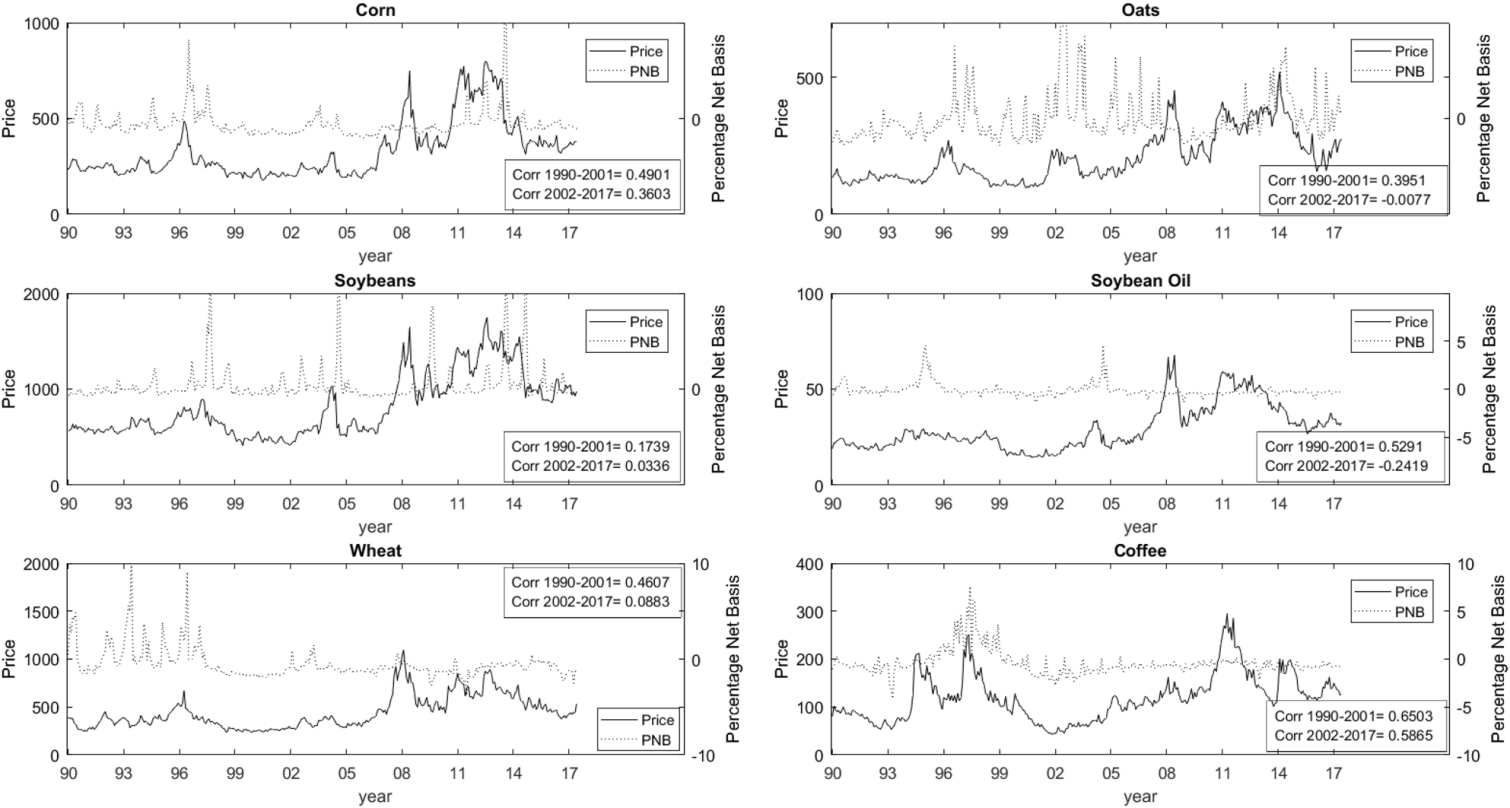
Commodity prices and percentage net basis

In Fig. 2, we compare commodity prices (solid line) with our measure of relative value, the PNB (broken line). For all cases, peaks in commodity prices are generally associated with higher levels of PNB. This is certainly the case for the first half of our sample. The link is not as strong for the second half of our sample. This point is further emphasized when we compare the correlation coefficients for pre and post 2002. The values for the PNB range between 5% and 10% (of the commodity price) per month. These values indicate the importance of the convenience yield, the benefit of holding the physical asset. They indicate that firms were willing to pay between 5% and 10% of the price to hold the commodity in its physical form.^12^ The link between commodity prices and PNB is not as strong for the second half of our sample and along with the findings from Fig. 1 point to a possible role for financialization and by extension speculation and manipulation.

### Empirical Results

**Fig. 3:**
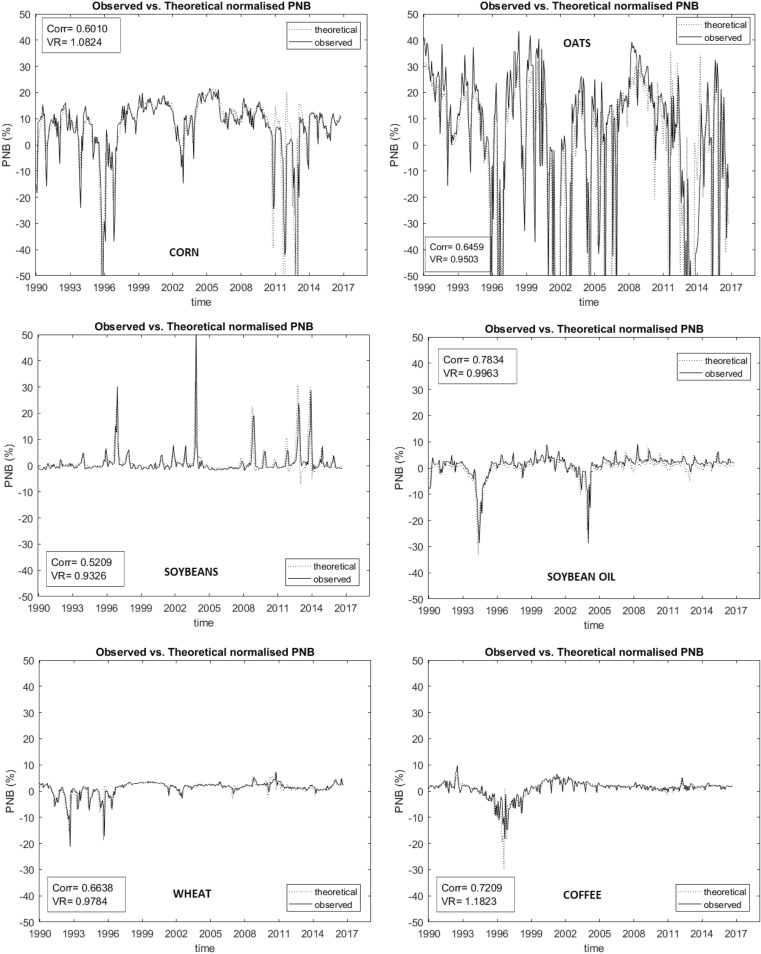
Commodities—Fundamentalists

In Fig. 3, we plot the relationship between the actual PNB (solid line) and the theoretical PNB (broken line) for all six commodities.^13^ In the figure, we also report two measures of fit between the theoretical and actual PNB, namely the correlation between the two series and their variance ratio. The correlation coefficient and the variance ratio represent an indication of the long-run and short-run proximity, respectively, between the actual PNB and the PNB determined by the commodity pricing model. As discussed earlier in the paper, a purely fundamentals-based approach is initially examined. All indications are that this model is not a suitable representation of prices. We then examine a behavioural model which incorporates Fundamentalists, Speculators and Manipulators.

**Fig. 4:**
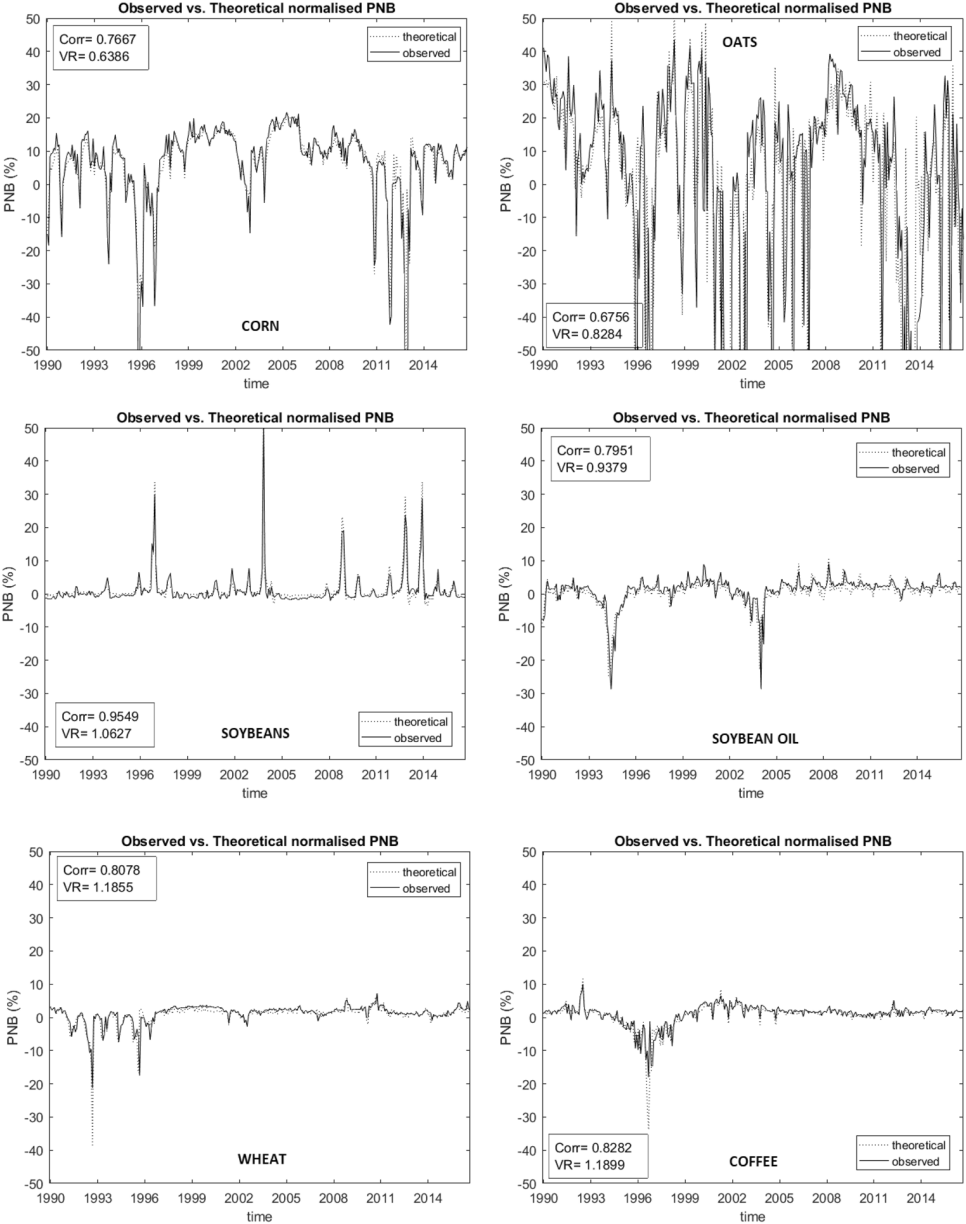
Commodities—Behavioral Model

The relationship between actual and theoretical PNB, for the case of the fundamentals-based model, are reported in Fig. 3. In the figure, the variance ratios are very close to one. While the correlations are consistently above 0.5, they are also significantly below 1. Although commodity prices would appear to be relatively aligned with movements in fundamentals, there is clearly some missing information which is reflected in the imperfect correlations between actual and theoretical PNB. The fact that the variance ratio is near unity means that this deviation does not necessarily disappear or does so slowly. The impact of both Speculators and Manipulators has the potential to explain this finding. In Fig. 4, we present our behavioral model, which now augments the Fundamentalists (highlighted above) with both Speculators and Manipulators. The correlation coefficients increase in all cases, though sometimes the variance ratios depart somewhat from unity. On balance, these results support the behavioural multi-agent setting, suggesting it can explain both long-run and short-run behaviour of commodity prices better than the classical fundamentals only model.

**Fig. 5:**
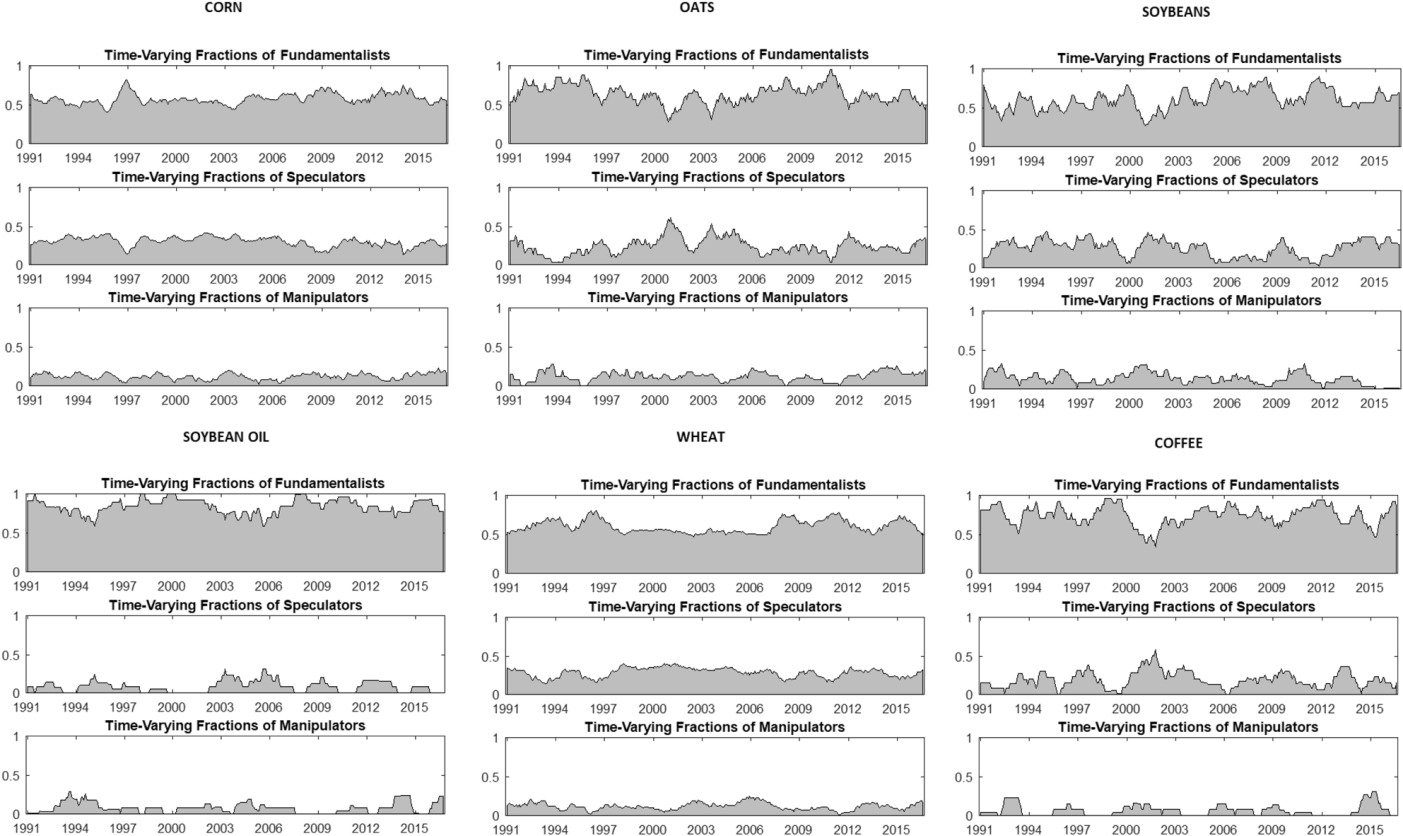
Commodities—Behavioral Model Weights

While the statistical performance of the behavioral model is certainly superior to the alternatives, the results presented to date do not indicate the role played by each set of actors in relation to the determination of commodity prices. We now turn to the specific weights associated with Fundamentalists, Speculators and Manipulators assuming the behavioral model is adopted. The weights are presented in Fig.  5 for all six commodities. With the exception of soybeans, all other commodities are heavily driven by Fundamentalists. The weights are well over 60% for the majority of the sample. Both Speculators and Manipulators play a consistent, yet relatively minor, role throughout our sample. Most importantly, there is no evidence that there is any association between Speculator/Manipulator activity and the financialization process which emerged around 2002.

The results presented in Fig. 5 point towards a significant and consistent role for speculation and manipulation. To shed further light on this, we formally test our hypothesis (Hypothesis 1) that the combined role of speculation and manipulation outweighs the role played by fundamentals over the course of our sample. Table 1 reports consistent evidence that the share of long-term versus short-term trading are not equal. In addition, we reject the null that short-term trading (speculation and manipulation) dominate long-term (or fundamentals-based) trading activity. This is consistently the case for all commodities examined. Our results also support previous evidence reported in the economics and finance literature, e.g. Sanders and Irwin (2011).

Our second hypothesis test, examines the extent of systematic short-term trading across our commodities. While we have determined that both forms of short-term (speculation and manipulation) trading do not dominate fundamentals, a popular narrative is that a wave of manipulation has occurred with the advent of financialization. In Table 2, we formally test whether manipulation is systematic across all commodities (Hypothesis 2). For each pair of commodities, the statistic (and the *p*-value) of a test of equality of the mean weights of the Manipulator (from the behavioral model) are reported. If a wave of manipulation had occurred, we would expect to find consistent mean weights for all six food commodities examined. Instead for 10 of the 15 pairs, we reject any equality in mean values. Again our results find no evidence of a wave of manipulation in food commodities.

Our final test examines the impact of financialization (if any) on manipulation in food commodities. This is not a formal test of financialization, but rather of whether there has been a notable change in manipulation during the period most associated with financialization. As indicated earlier in the paper, we draw on Chari and Christiano (2017) and take 2002 as the starting point of the financialization process.^14^ In Table 3, we report hypothesis test results examining whether manipulation is distributed consistently across our pre and post financialization periods (Hypothesis 3). For two of the six commodities, we reject the null hypothesis of equality of weights between pre and post ‘financialization’. In terms of our one tailed test, it is only wheat that indicates higher manipulation weights in the financialization period.

### CFTC Implied Weights

As a sensitivity test, we also implement our hypothesis tests using the weights of different agents obtained directly from CFTC data, rather than our behavioural model. This data is sourced from ‘Commitment of Traders’ (COT) reports. These reports display the total open interest of each commodity and their profile of traders. Clearing members, futures commission merchants and reporting firms, file daily reports with the CFTC. It is estimated that approximately 70 to 90% of the total open interest in any commodity is reported to the CFTC (Chari and Christiano 2017). We use information on these reported traders to infer the weights of different agents.

When an individual reportable trader is identified by the CFTC, her trades are classified either as ‘commercial’ or ‘non-commercial’. Trades that use futures contracts for hedging purposes are defined as ‘commercial’.^15^ To ensure that traders are classified with accuracy and consistency, CFTC staff may exercise judgment in re-classifying a trader if there is additional information about how the trader uses the markets. To make use of the CFTC open interest data, we thus propose a complementary definition of the three agents from our behavioral model which maps them to the types of trader considered by the COT reports.

The long-term traders are defined as the percentage of trades opened exclusively by ‘commercial’ traders. That is, when both the long position and the short position in a trade is being held by ‘commercial’ traders. We approximate this percentage as the share that represents the minimum between the total long position and total short position held by commercial traders. The short-term traders are defined as the percentage of trades over the total open interest where ‘non-commercial’ traders participate. This category represents the short-term investors highlighted in our behavioural model. Consistent with our behavioural model, we now distinguish between two types of short-term traders. The Speculators is the first type, where the long (short) position of the trade is held by a commercial agent and the short (long) position is held by a non-commercial trader. We approximate this by the share (of total trades), represented by the difference between the reported positions of commercial and non-commercial traders. Finally, Manipulators are defined as the percentage of trades opened exclusively by ‘non-commercial traders’. We approximate this by the share (of total trades) represented by the minimum between the total long position and total short positions held by non-commercial traders.^16^

Table 4 reports consistent results with those found using our behavioural model. Both the share of long-term versus short-term trading are not equal and we reject the null of Hypothesis 1 that short-term trading (speculation and manipulation) dominate long-term (or fundamentals) based trading activity. This is the case for all commodities examined. Again, we find very little evidence of a wave of manipulation occurring during the period most associated with the advent of financialization. The results for Hypothesis 2 are reported in Table 5. Finally in Table 6, we report the hypothesis results examining whether manipulation is distributed consistently across our pre- and post-financialization period (Hypothesis 3) using the alternative CFTC approach. We do find much stronger evidence of a difference in behaviour pre and post financialization. In addition, for the majority of commodities examined, the indications are that manipulation has increased post 2002.

**Table 5:** Manipulators and pairwise commodities—CFTC

Statistic (*p* value)	Corn	Oats	Soybeans	Soybean oil	Wheat
Oats	12.7708***				
	(0.0000)				
Soybeans	− 1.8429	− 17.2741***			
	(0.0658)	(0.0000)			
Soybean oil	− 4.3630***	− 18.6404***	− 3.0526***		
	(0.0000)	(0.0000)	(0.0000)		
Wheat	− 10.4643**	− 23.0813***	− 9.9469***	-6.9470***	
	(0.0024)	(0.0000)	(0.0000)	(0.0000)	
Coffee	− 3.0058**	− 15.8117***	− 1.5940	1.1050	7.5017***
	(0.0028)	(0.0000)	(0.1114)	(0.2696)	(0.0000)

This table shows the statistic and the *p*-values of a two-tailed equality mean tests between the weights of the Manipulator agent obtained from CFTC data for each pair of commodities. For instance, the intersection between the corn column and the oats row shows the test results for the Manipulator agent weights of corn and oats

***, ** and * represents rejection of the null hypothesis at 1%, 5% and 10% significance level after Bonferroni correcting the significance levels for multiple comparisons

**Table 6:** Financialization and manipulation—CFTC

Statistic (*p* value)	Corn	Oats	Soybeans	Soybean oil	Wheat	Coffee
$H_0: \mu ^{pre02}_{M}= \mu ^{pos02}_{M}$	$-15.2399$***	$-2.9657$***	$-10.4162$***	$-11.0042$***	16.4529**	$-18.0914$
	(0.0000)	(0.0000)	(0.0000)	(0.0000)	(0.0000)	(0.0000)
$H_0: \mu ^{pre02}_{M}< \mu ^{pos02}_{M}$	$-15.2399$	$-2.9657$	$-10.4162$	$-11.0042$	$-16.4529$	$-18.0914$
	(1.0000)	(0.9984)	(1.0000)	(1.0000)	(1.0000)	(1.0000)

This table shows the statistic and the *p*-values of a two-tailed equality mean tests between the weights of the Manipulator agent obtained from CFTC data before 2002 and weights of the Manipulator agent obtained from CFTC data after 2002 for each commodity

***, ** and * represents rejection of the null hypothesis at 1%, 5% and 10% significance level

## Conclusion

Drawing on Angel and McCabe (2009), we examine the role of a combined set of actors in relation to food price movements. We specifically examine Fundamentalists, Speculators and Manipulators. We adopt a behavioural model and data from traders to formally examine the role of speculation in food commodities during the period of financialization. Following Angel and McCabe (2009) we quantify the role played by ’good’ speculators (Speculators), those that push prices closer to the fundamental value and ’bad’ speculators, those that push prices away from the fundamentals (Manipulators).

Our results indicate that Speculators and Manipulators play a consistent, yet relatively minor, role throughout our sample. A sample that has become most associated with the process of financialization. Although there is no evidence of a wave of manipulation occurring, we do find results that point to an increased role played by Manipulators during the period most associated with financialization. Rather than relying purely on our behavioural model, we also implement our hypothesis tests using the weights of different agents obtained directly from CFTC ’Commitment of Traders’ data. Consistent results are found when using CFTC data, to those reported using our behavioural model.

Following Angel and McCabe (2009), ours is the first known study to formally quantify the role of Fundamentalists, Speculators and Manipulators for commodities in general and food commodities in particular. Our study clearly highlights the importance of identifying the share associated with each of these agents individually and over time. While manipulation certainly exists in the food commodity sector, there has been no evidence of a wave of manipulation during the recent period of financialization. Rather than rely solely on our behavioural model, we also examine trader behaviour within the industry. The ’Commitment of Traders’ data fully supports our findings. Finally, our results point to two approaches that can be adopted to monitor manipulative behaviour within food prices and can be used to inform the need for regulatory interventions.

## Appendix 1: Commodity Pricing Methodology

The commodity rate of return is defined as:

4 $$\begin{aligned} q_t = (P_{t+1} - P_t + \psi _t)/P_t \end{aligned}$$

The commodity return can be re-written purely in terms of the convenience yield and the percentage net basis (PNB). Following linearization and taking the conditional expectation, the solution to the difference equation can be defined as;

5 $$\begin{aligned} y'^F_t \equiv \sum ^\infty _{j=0} \beta ^jE_t(\beta q_{t+j} - \Delta \psi '_{t+j}) \end{aligned}$$

where the F represents fundamentals.^17^ The above is the definition for the PNB, when the price is set by a rational agent, with a long-run (infinite) horizon.

Besides the long-term solution detailed above, we can also obtain a short horizon (one period) alternative. The short-run (one-period rather than infinite horizon) counterpart of (5) is;

6 $$\begin{aligned} \begin{aligned} y'^{S}_t&= \frac{\beta }{1-\beta } \left( E_t(q_t) - E_t \left( \frac{\Delta P_{t+1}}{P_t} \right) \right) - E_t (\Delta \psi '_t) \end{aligned} \end{aligned}$$

The approximate equality in (6) is similar to the *Fundamentalist* model, with the difference that the price is now set by a rational agent with a *short-run* horizon. We therefore refer to it as the *Speculator* (*S*) model of the PNB.

In the model in which the representative investor is the *Fundamentalist*, namely (5), the normalized PNB is a weighted average, with geometrically declining weights, of the expected return adjusted for the one period expected change in the convenience yield. Instead, in the model with the *Rational Speculator* (RS) representative investor, namely (6), the normalized PNB is proportional to the difference between expected return and the expected rate of change of the commodity price, adjusted for the one period expected change in the normalized convenience yield. Therefore, while expected changes in the convenience yield and rates of return play a role in both the short-run and long-run model, the expected rate of price change (capital gain) plays a direct role (and with a large weight) only in the short-run model.

We also consider the case that prices are influenced by a short-horizon irrational contrarian, what we refer to as manipulators;

7 $$\begin{aligned} \begin{aligned} y'^{M}_t&= \frac{\beta }{1-\beta } \left( E_t(q_t) + E_t \left( \frac{\Delta P_{t+1}}{P_t} \right) \right) - E_t (\Delta \psi '_t) \end{aligned} \end{aligned}$$

We refer to (7) as the *Manipulator* (*M*) model of the PNB. The PNB in this case is proportional to the sum of the expected return and the expected rate of change of the commodity price, rather than their difference as in the case of the model (*S*) with the rational short-horizon marginal agent, adjusted for the one period expected change in the normalized convenience yield. A complete description of the methodology along with formal definitions are detailed in Bredin et al. (2018).

## Appendix 2: Commodity Data

See Table 7.

**Table 7:** Commodity contracts

Commodity	Exchange	Delivery months
Corn	CBOT	Mar, May, Jul,Sep, Dec
Oats	CBOT	Mar, May, Jul, Sep, Dec
Soybeans	CBOT	Jan, Mar, May, Jul, Aug, Sep, Nov
Soybean oil	CBOT	Jan, Mar, May, Jul, Aug, Sep, Oct, Dec
Wheat	CBOT	Mar, May, Jul, Sep, Dec
Coffee	ICE	Mar, May, Jul, Sep, Dec

Chicago Board of Trade (CBOT), Intercontinental Exchange (ICE).

## Footnotes

1 In addition, as highlighted by Smith (2014) pretty much all decisions that are made by producers, merchants and consumers incorporate some form of speculation. Clearly merchants engage in speculation when determining what they are prepared to pay now versus what the commodity will be worth in the future.

2 Gorton and Rouwenhorst (2006) have highlighted that commodity futures allow producers to hedge their exposure to uncertain price movements in the future. In return for providing this insurance, the investors (or Speculators) in commodity futures are paid a compensation for bearing this risk. This transfer of risk from producers to Speculators reflects the socially beneficial role being played by Speculators. However, with Manipulators pushing prices away from their fundamental value, prices do not reflect the proper information signal to consumers and producers. We thank an anonymous referee for highlighting this point.

3 See Bank of International Settlements, https://www.bis.org/.

4 Since 2011, negative commodity returns have meant that the financial headlines have been less enthusiastic, with references to little or no interest in commodities from the investment community. See Meyer and Authers (2015).

5 Hamilton and Wu (2014) highlight 2005 as a structural break in relation to commodity prices.

6 Information regarding market conditions is reflected in the relationship between futures prices at different maturities, the term structure of commodity prices. In markets with scarce supply of the physical commodity, close to expiring contracts will trade at a higher price. This is referred to as backwardation. While for scenarios when we have surplus inventory, the reverse will be the case. This is referred to as contango. Considerable evidence exists to indicate that food-based commodities tend to spend relatively similar amounts of time in backwardation or contango, see Feldman and Till (2006). A sustained period of contango during the financialization period could reflect a sustained period of speculative activity driving prices. However, evidence presented by Feldman and Till (2006) indicate that there is no evidence of sustained contango throughout the late 1990s and into the 2000s.

7 As is the convention in the literature, see Pindyck (1993), we model the difference equation. A detailed description is provided in the Appendix 1, including a solution to the difference equation. The normalized PNB for the case of *F*, *S*,  and *M* are also derived in detail in Appendix 1.

8 It is important to note that no clear consensus exists in the literature, as to whether individual commodities or indexes of commodities have been subject to financialization. Studies that have found in favour of financialization in commodities include; Henderson et al. (2015), Singleton (2014) and Tang and Xiong (2012). While studies finding evidence against include; Irwin and Sanders (2011) and Stoll and Whaley (2010). The lack of a consensus extends to the literature on price discovery, with Dimpfl et al. (2017) emphasising the importance of the spot market, while Bohl et al. (2020) highlighting the role of the futures markets, as a result of speculative activity. Separately, McLean and Pontiff (2016) have highlighted evidence of a systematic link between the disappearance of anomalies and the publication of academic papers.

9 It is important to emphasize we do not formally test for financialization in our study. Our interest in financialization (if it exists) is how it influences manipulation.

10 A complication that can exist when examining commodities, is that a ’spot’ (or futures) contract may not trade in every month for all commodities. For those months when a ’spot’ contract does not trade, the standard approach is to use a linear interpolation to determine the ’spot’ contract price. See Pindyck (1993).

11 Commodity index traders are some times referrred to as index speculators.

12 This is in excess of the standard costs of holding the physical commodity, the cost of carry. These costs would include interest rate and storage costs.

13 The theoretical PNB is calculated using a forecasting system of equations which is derived from Eq. 1, using a vector autoregression model. Details on the methodology along with formal definitions are described in Bredin et al. (2018). The normalized PNB is adopted in the empirical modelling.

14 As has already been indicated, Hamilton and Wu (2014) highlight 2005 as a structural break (linked to financialization) in relation to commodity prices. We find consistent results when using 2005 as the starting point of the financialization process.

15 See CFTC Regulation 1.3, 17 CFR 1.3(z). A trading entity generally gets classified as a ‘commercial’ trader by filing a statement with the CFTC, namely the “CFTC Form 40: Statement of Reporting Trader”, that it is commercially “*...engaged in business activities hedged by the use of the futures or option markets.*”

16 The direct link to the commercial trader, is consistent with the transfer of risk from producers to Speculators and reflects the socially beneficial role being played by Speculators. Manipulators are defined as the residual share of short-term traders and have no such link to producers. While Speculators will push prices closer to their fundamental value, Manipulators will push prices away from their fundamental value.
